# Galileo and BeiDou AltBOC Signals and Their Perspectives for Ionospheric TEC Studies

**DOI:** 10.3390/s24196472

**Published:** 2024-10-08

**Authors:** Chuanfu Chen, Ilya Pavlov, Artem Padokhin, Yury Yasyukevich, Vladislav Demyanov, Ekaterina Danilchuk, Artem Vesnin

**Affiliations:** 1 Faculty of Physics, Lomonosov Moscow State University, 119991 Moscow, Russia; chuanfu.chen@physics.msu.ru (C.C.); padokhin@physics.msu.ru (A.P.); 2 Pushkov Institute of Terrestrial Magnetism, Ionosphere and Radiowave Propagation RAS, 108840 Moscow, Russia; 3 Institute of Solar-Terrestrial Physics SB RAS, 664033 Irkutsk, Russia; sword1971@yandex.ru (V.D.); danilchuk.k@mail.ru (E.D.); artem_vesnin@iszf.irk.ru (A.V.)

**Keywords:** AltBOC, TEC, BPSK, QPSK, BeiDou, Galileo, GNSS, IGS, radio frequency interference

## Abstract

For decades, GNSS code measurements were much noisier than phase ones, limiting their applicability to ionospheric total electron content (TEC) studies. Ultra-wideband AltBOC signals changed the situation. This study revisits the Galileo E5 and BeiDou B2 AltBOC signals and their potential applications in TEC estimation. We found that TEC noises are comparable for the single-frequency AltBOC phase-code combination and those of the dual-frequency legacy BPSK/QPSK phase combination, while single-frequency BPSK/QPSK TEC noises are much higher. A two-week high-rate measurement campaign at the ACRG receiver revealed a mean 100 sec TEC RMS (used as the noise proxy) of 0.26 TECU, 0.15 TECU, and 0.09 TECU for the BeiDou B2(a+b) AltBOC signal and satellite elevations 0–30°, 30–60°, and 60–90°, correspondingly, and 0.22 TECU, 0.14 TECU, and 0.09 TECU for the legacy B1/B3 dual-frequency phase combination. The Galileo E5(a+b) AltBOC signal corresponding values were 0.25 TECU, 0.14 TECU, and 0.09 TECU; for the legacy signals’ phase combination, the values were 0.19 TECU, 0.13 TECU, and 0.08 TECU. The AltBOC (for both BeiDou and Galileo) SNR exceeds those of BPSK/QPSK by 7.5 dB-Hz in undisturbed conditions. Radio frequency interference (the 28 August 2022 and 9 May 2024 Solar Radio Burst events in our study) decreased the AltBOC SNR 5 dB-Hz more against QPSK SNR, but, due to the higher initial SNR, the threshold for the loss of the lock was never broken. Today, we have enough BeiDou and Galileo satellites that transmit AltBOC signals for a reliable single-frequency *vTEC* estimation. This study provides new insights and evidence for using Galileo and BeiDou AltBOC signals in high-precision ionospheric monitoring.

## 1. Introduction

The ionosphere plays a crucial role in various practical applications including radio communication and sounding, as well as Global Navigation Satellite Systems (GNSSs) such as GPS, GLONASS, Galileo, and BeiDou. This highly ionized region of the Earth’s atmosphere, extending from about 60 km to 1000 km above the Earth’s surface, is responsible for refraction, diffraction, and scattering radio waves, which directly impacts the signal propagation used in GNSS. The ionosphere’s variable nature, influenced by forcing from above, e.g., solar radiation and geomagnetic activity, and below, e.g., atmospheric and lithospheric sources, can cause significant disturbances to GNSS signals, leading to errors in positioning and navigation.

These errors are primarily due to ionospheric delays, which vary with time, location, and solar and geomagnetic conditions. Thus, the reliable monitoring of the ionospheric variability at different spatial and temporal scales becomes very important. One of the important parameters here is the total electron content (TEC), which is a columnar number of electrons in the ionosphere for the considered line of sight.

Researchers often use the TEC for studies that need measuring fine effects [1,2,3]. The TEC is used as an input parameter for different techniques: radio tomography [4,5], radio interferometry of traveling ionospheric disturbances (TID) [6,7], and global [8,9,10] and regional [11,12] TEC mapping. Space weather applications use the TEC [13,14,15] to calculate various indices of ionospheric disturbance [16,17,18]. Researchers use TEC datasets to develop empirical models [19], and estimate the accuracy of models [20,21]. There are ionospheric models that can assimilate TEC data [22,23].

Today, GNSSs offer worldwide coverage and a high time resolution to estimate the TEC. This involves signal phase delay and pseudorange (group delay) observations [24]. There are two approaches to estimating the TEC from GNSS observations. The dual-frequency approach exploits the frequency dependence of the ionospheric delay. The single-frequency approach involves different signs in ionospheric delays for phase range and pseudorange. Using traditional modulation schemes—binary-phase shift keying (BPSK), quadrature-phase shift keying (QPSK), and binary offset carrier (BOC) [25]—the pseudorange noises exceed the phase noises by an order. That results in huge noises in the single-frequency raw slant TEC compared to ones in the dual-frequency phase TEC. This increased noise restricts the use of the single-frequency TEC, except for certain data from geostationary satellites [26] and lower-grade GNSS receivers [27].

Another crucial issue with legacy GNSS signals is their performance under the conditions of radio frequency interference (RFI), for example, caused by solar radio bursts (SRBs) [28,29], when the SNR can substantially drop below the 25–30 dB-Hz threshold, significantly increasing number of losses of locks (LoLs). To address these challenges, there is a pressing need to improve GNSS signals. This includes adopting more advanced modulation techniques, such as Alternative Binary Offset Carrier (AltBOC), which enhances the signal robustness against interference and improves the performance in multipath environments [30]. Additionally, increasing the signal power and employing frequency diversity can effectively reduce the probability of LoL, thereby enhancing the overall reliability and accuracy of GNSS systems.

Recently, Galileo and BeiDou systems started to exploit extra-wideband E5 and B2 AltBOC signals [30] available with modern geodetic receivers. Padokhin et al. [31] reports that Galileo E5 has a comparable level of noise to the dual-frequency phase combination level of noise in the single-frequency phase-code TEC estimate and increased the SNR compared to legacy signals.

The current article studies the potential of BeiDou B2 AltBOC signals for TEC estimates and compares it with Galileo E5. For that, we compare the TEC noises when different observables are used. We also study the possibility of absolute vertical TEC estimation using solely single-frequency AltBOC data. We further consider the performance of the Galileo E5 and BeiDou B2 signals under conditions of RFI caused by a solar radio burst on 28 August 2022.

The article is organized as follows: Section 2 gives a brief description of the Galileo E5 and BeiDou B2 AltBOC signals. It is followed by Section 3, which focuses on the single-frequency TEC and vertical TEC estimations and TEC noise proxy used in current research. Section 4 describes the experimental layout and data. Section 5 provides examples of the single-frequency AltBOC TEC and absolute vertical TEC estimations and statistics of the TEC noise for the whole studied period. Section 6 discusses the AltBOC signals’ performance under RFI conditions during the SRBs on 28 August 2022 and on 9 May 2024. Section 7 provides the main findings of the research.

## 2. Galileo E5 and BeiDou B2 AltBOC Signals

AltBOC coding offers significant advantages over traditional BPSK and QPSK modulation schemes in GNSSs. It provides a superior performance in terms of timing accuracy, interference resistance, and overall signal robustness. AltBOC modulation divides the carrier spectrum into positive and negative frequency bands, with each band modulating signals using different phases, resulting in a higher spectral efficiency. In contrast to BPSK, where each symbol carries one bit of information, and QPSK which carries two bits per symbol, AltBOC can transmit multiple signals simultaneously across a wider bandwidth, thereby carrying more information per unit of spectrum, making it particularly advantageous in environments where frequency resources are limited [30,32].

**Figure 1:**
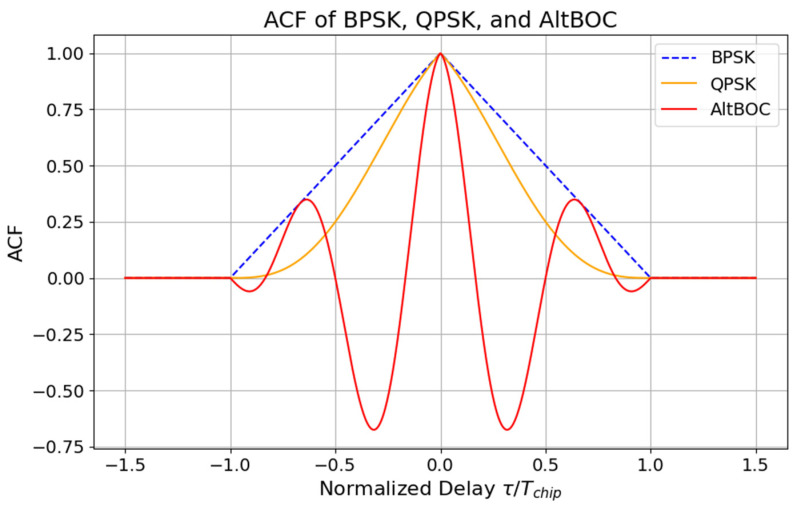
Autocorrelation functions for BPSK, QPSK, and AltBOC signals.

AltBOC modulation also exhibits significant advantages in resisting multipath interference, owing to its complex autocorrelation function (ACF) structure. Figure 1 shows ACFs for signals with different modulation schemes, in particular, BPSK, QPSK, and AltBOC. As observed, AltBOC’s ACF has the sharpest main peak with lower side lobes, indicating its superior ability to distinguish between direct and reflected signals in multipath environments, thus reducing errors. The multipath peaks in AltBOC’s autocorrelation function provide the receiver with a higher delay resolution, which is crucial for accurate positioning.

**Figure 2:**
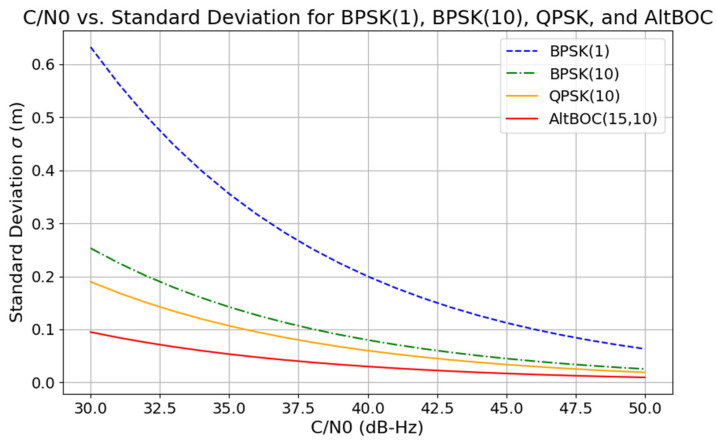
Pseudorange noises for BPSK, QPSK, and AltBOC signals.

Since the code tracking noise is inversely proportional to the steepness of the ACF, this should result in a decrease in the pseudorange noise for th eAltBOC signals compared to the BPSK and QPSK ones. This can be seen in Figure 2. AltBOC outperforms both BPSK and QPSK signals with a pseudorange noise of below 5 cm for an SNR greater than 35 dB-Hz, highlighting its superiority in signal accuracy and robustness. Theoretical estimates presented in Figure 1 and Figure 2 are obtained with typical GNSS coding parameters, integers (m, n) and (n) in brackets stand for multipliers for the subcarrier frequency $f_{s}=m\times f_{0}$ and chip rate frequency $f_{chip}=n\times f_{0}$, with $f_{0}$ = 1.023 MHz, and $T_{chip}$ is the chip length which depends on the coding scheme. Note that the pseudorange noise will directly affect any TEC estimation method involving code measurements.

Furthermore, AltBOC reduces the cross-correlation interference between different PRN codes by separating the spectral positions of different signals. The spectral separation and subcarrier modulation enhance the interference resistance and improve the signal reception reliability [30].

Although AltBOC’s structure is complex and its receiver implementation is more costly, its comprehensive advantages in multipath resistance, noise resistance, delay resolution, and spectral efficiency make it the superior choice for GNSS systems requiring high-precision positioning.

The design of the BeiDou B2 and Galileo E5 signals fully considers these factors, adopting AltBOC modulation to deliver a higher accuracy and reliability in positioning services. Table 1 below summarizes the main characteristics of the Galileo E5 and BeiDou B2 signals.

Both Galileo E5 and BeiDou B2 signals employ the AltBOC (15,10) modulation format, with a central frequency of 1191.795 MHz, covering the E5a (or B2a for BeiDou) 1176.45 MHz and E5b (or B2b for BeiDou) 1207.14 MHz frequencies. This combination extends the signal’s bandwidth to approximately 51.15 MHz and significantly enhances the accuracy in multipath environments. This design ensures the signal’s wideband characteristics and enhances its performance in challenging environments, making the BeiDou B2 signal a critical component for high-precision services.

Both the Galileo E5 and BeiDou B2 signals incorporate advanced technical features to meet the high precision and reliability demands of modern GNSS applications. Their dual-frequency capabilities and complex spectral structures make them indispensable in navigation and positioning services, catering to a wide range of applications from civilian to professional use. AltBOC (15,10) is particularly significant in modern GNSS systems for its dual-frequency capabilities, wideband characteristics, and complex spectral structure, which are crucial for achieving a high precision and reliability.

## 3. Ionospheric TEC Estimation Using Single-Frequency Code—Phase Combination

The ionospheric TEC estimation is a critical aspect for understanding and mitigating ionospheric effects on GNSS signals. This estimation is commonly performed using combinations of the signal frequency code (*P*) and phase (*L*) measurements.

The ionosphere contributes the phase and the group refractivity index oppositely, so the combination provides the single-frequency slant *TEC* [12]:

(1) $$sTEC=\frac{f_{i}^{2}}{2K}\left(P_{i}-\frac{L_{i}c}{f_{i}}\right)+const$$

where *K* = 40.308 $m^{3}/s^{2}$, *c* is speed of light, and $f_{i}$ is an operating frequency.

For the sake of completeness, we provide here the dual-frequency phase and code combinations for the *TEC* estimations, which would serve as benchmarks in our study [24].

(2) $$sTEC=\frac{c}{K}(\frac{L_{i}}{f_{i}}-\frac{L_{j}}{f_{j}})\frac{f_{i}^{2}f_{j}^{2}}{f_{i}^{2}-f_{j}^{2}}+const$$

(3) $$sTEC=\frac{1}{K}(P_{j}-P_{i})\frac{f_{i}^{2}f_{j}^{2}}{f_{i}^{2}-f_{j}^{2}}+{DCB}_{ij}$$

where $f_{i}$ and $f_{j}$ are GNSS operating frequencies depending on the system, and ${DCB}_{ij}$ stands for the sum of differential code biases in satellites transmitting and receivers receiving chains. Note that, like the dual-frequency phase combination (2) or dual-frequency code combination (3), the single-frequency combination (1) also provides a relative *TEC* estimate due to the unknown initial phase value.

The article mainly focuses on the raw *sTEC* data quality. We used the 100 s *TEC* root-mean-square (100 s *TEC RMS*) as a quality metric (indicator of *TEC* noise) [31]:

(4) $$TEC\;RMS=\sqrt{<TEC^{2}{>}_{100s}-<TEC{>}_{100s}^{2}}$$

To choose the interval length, we used the following reasons. First, we need a long interval to calculate statistically significant estimates. Second, we cannot use an interval that is quite long to avoid satellite motion and ionospheric variability effects. Third, using an interval of 100 s will allow us to compare the results with [31].

The relative slant *TEC* estimations obtained using (1) or (2) could serve as the input data for the single-station absolute vertical TEC estimation. Note that we do not use combination (3) due to its significant noise level. This combination is typically used in approaches involving phase/code smoothing and leveling and introduces the problem of DCB estimation. There are a number of methods for single-station *vTEC* estimations but they generally require either a DCB or unknown phase constant estimation, which, in turn, require additional assumptions.

We used an algorithm that involved the phase-difference approach that is often used in ionospheric radio tomography [33], so the DCB or constants are excluded by rearranging the input equations. The algorithm uses the *vTEC* model representation in the vicinity of the station as a truncated series of Taylor expansions up to the second order in space and time:

(5) $$\begin{array}{cc} vTEC=a_{0} & +a_{1}(lat_{IPP}-lat_{st})+a_{2}(lat_{IPP}-lat_{st}{)}^{2}+\\ & +a_{3}(lon_{IPP}-lon_{st})+a_{4}(lon_{IPP}-lon_{st}{)}^{2}+\\ & +a_{5}(t_{obs}-t_{est})+a_{6}(t_{obs}-t_{est}{)}^{2}+\\ & +a_{7}\left(lon_{IPP}-lon_{st}\right)\left(lat_{IPP}-lat_{st}\right)+\\ & +a_{8}\left(lon_{IPP}-lon_{st}\right)\left(t_{obs}-t_{est}\right)+\\ & +a_{9}\left(lat_{IPP}-lat_{st}\right)\left(t_{obs}-t_{est}\right) \end{array}$$

where ${lat}_{IPP}$, $lon_{IPP}$ are the latitude and longitude of the ionospheric piercing point (IPP), $lat_{st}$, $lon_{st}$ are the latitude and longitude of the station (receiver), $t_{obs}$, $t_{est}$ are the time of the current observation and the time moment of the *vTEC* estimation, and {$a_{i}$} are 10 coefficients that need to be estimated. Only observations which fall within $\left|t_{obs}-t_{est}\right|$ < 15 min contribute to these coefficients. For the vertical *vTEC* to slant *sTEC* conversion, we use a single-layer mapping function:

(6) $$\begin{array}{c} sTEC=MF\cdot vTEC\\ MF=\frac{1}{\sqrt{1-{\left(\frac{R_{E}\cdot cos(el)}{R_{E}+h_{IPP}}\right)}^{2}}} \end{array}$$

where *el* is the elevation angle of the satellite, $h_{IPP}$ is the height of the IPP (450 km), and $R_{E}$ is the Earth’s radius.

Equations (5), (6), and (1) or (2), for all satellites in view, form a system of linear equations (SLE) to estimate {$a_{i}$}. Following [10], the algorithm for *vTEC* involves the so-called phase-difference approach: for each satellite receiver pair during the continuity interval between consecutive losses of phase locks, the constants in (1) and (2) remain the same and could be successfully removed by the pairwise subtraction of equations in the system. Such a procedure is applied for each continuity interval for each satellite receiver pair. For example, if the single-frequency combination (1) is considered as the input data, it can be written as follows:

(7) $${sTEC}^{k}\left(t_{j}\right)-{sTEC}^{k}\left(t_{j-1}\right)=\frac{f^{2}}{2K}\left(P^{k}\left(t_{j}\right)-P^{k}\left(t_{j-1}\right)-\frac{c}{f}\left(L^{k}\left(t_{j}\right)-L^{k}\left(t_{j-1}\right)\right)\right)={MF}^{k}\left(t_{j}\right){vTEC}^{k}\left(t_{j}\right)-{MF}^{k}\left(t_{j-1}\right){vTEC}^{k}\left(t_{j-1}\right)=a_{0}\left({MF}^{k}\left(t_{j}\right)-{MF}^{k}\left(t_{j-1}\right)\right)+\;a_{1}\left({{MF}^{k}\left(t_{j}\right)lat}_{IPP}^{k}\left(t_{j}\right)-{MF}^{k}\left(t_{j-1}\right){lat}_{IPP}^{k}\left(t_{j-1}\right)-lat_{st}\left({MF}^{k}\left(t_{j}\right)-{MF}^{k}\left(t_{j-1}\right)\right)\right)+\ldots +a_{5}\left({MF}^{k}\left(t_{j}\right)t_{j}-{MF}^{k}\left(t_{j-1}\right)t_{j-1}-t_{est}\left({MF}^{k}\left(t_{j}\right)-{MF}^{k}\left(t_{j-1}\right)\right)\right)+\ldots$$

where *k* stands for the satellite and *j* for the different time moments ($t_{obs})$.

The resulting system can be easily solved for {$a_{i}$} using the least squares method, in which we additionally apply weights of observations depending on its elevation angle as described in [10].

To ensure positive *vTEC* values, we use least squares with constraints implemented by solving the corresponding liner complementarity problem (LCP) as described in [10]. The program realization of this algorithm is publicly available via GitHub [34]. In the next section, we will also consider the possibility of estimating the single-station absolute *vTEC* based on the single-frequency phase/code combination (1) for AltBOC signals by applying the algorithm described above.

## 4. Experimental Data

To investigate the influence of the coding scheme on the single-frequency TEC estimations and their noises, one should consider high-rate (of at least 1 Hz sampling) observations from receivers capable of tracking both AltBOC signals and legacy BPSK and QPSK ones. For Galileo E5(a+b), choosing such a receiver is not a problem. For example, more than two-thirds of EUREF stations are able to track these signals [35]. With the recently introduced BeiDou B2(a+b) signal, the situation is, up until now, not so favorable. Only a few of the receivers in publicly available networks are capable of tracking it. Thus far, in our study, we considered two of such receivers from the IGS network providing high-rate data for both the Galileo E5(a+b) and BeiDou B2(a+b) signals. Table 2 presents the detailed information about these stations.

These receivers are located in different latitudinal and longitudinal regions, the equatorial African sector for ACRG and the midlatitude American sector for SGPO, providing the opportunity to conduct the study in different geophysical environments. Note also that both stations are equipped with choke-ring-type antennae, which provide good multipath mitigation. Both stations are equipped with the same type of Javad receiver [36], which guarantees, to some extent, the equivalent processing of signals.

We considered a two-week period of observations, 14–29 February 2024, to obtain reasonable statistics of the TEC noises. The geomagnetic conditions during the observation period according to WDC for Geomagnetism, Kyoto [37] were undisturbed. The minimum Dst index did not exceed −30 nT, and the Kp index was typically below 3, reaching peak values of 4+ on 27 February 2024.

To study the AltBOC signals’ performance under the conditions of strong RFI, we additionally considered data for 28 August 2022, when intense SRBs occurred at ~17:45 UT and seriously affected GNSS stations in American sector [29]. The next two sections describe the results obtained in our study.

## 5. Single-Frequency AltBOC TEC and TEC Noise

**Figure 3:**
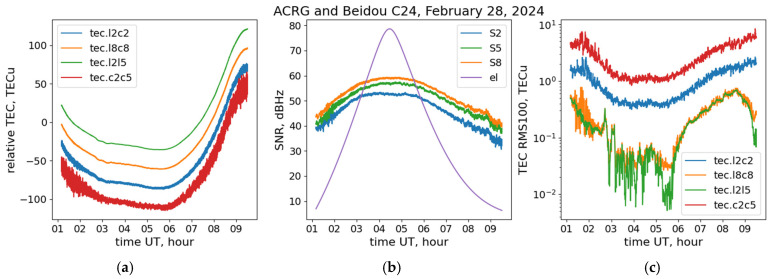
Slant TEC (**a**), SNR (**b**), and TEC RMS (**c**) for the ACRG-BeiDou C24 pass on 28 February 2024. In panels (**a**,**c**), the green, orange, blue, and red lines correspond to L2L5, L8C8, L2C2, and C2C5 combinations, correspondingly. In panel (**b**), the orange, green, and blue solid lines correspond to S8, S5, and S2 observables, correspondingly. The purple line in panel (**b**) shows the satellite’s elevation angle.

This section presents examples of case studies of the single-frequency TEC estimation and TEC noises obtained with the Galileo and BeiDou AltBOC signals on 28 February 2024 at the ACRG receiver, as well as the whole statistics of the TEC noises at this station during the entire studied period. Figure 3b shows the SNR of different signal components from the BeiDou C24 satellite at the ACRG station. It is obvious that all three observables demonstrate similar behavior with an increasing SNR with the increasing satellite elevation angle (also shown in the panel). At the same time, the SNR S8 for the B2(a+b) AltBOC signal is greater than the SNR S2 for the B1I BPSK(2) signal by ~7.5 dB-Hz, and by ~2.5 dB-Hz than the SNR S5 for the QPSK(10) B2a signal throughout the whole pass.

Figure 3a shows the relative slant TEC estimations via the (1)–(3) combinations, obtained at this satellite–receiver pair. We can see that the dual-frequency pseudorange combination (3) constructed with the C2 BPSK(2) and C5 QPSK(10) observables possesses the largest amount of noise among all considered TEC combinations, especially at low elevation angles. The single-frequency code/phase combination (1) built upon the L2 and C2 BPSK(2) observables is slightly less contained by the noise, but its level still remains significant. The best noise figure, as expected, shows the dual-frequency phase combination (2) built upon the L2 BPSK(2) and L5 QPSK(10) observables. However, it is important that a comparable level of noise shows the single-frequency code/phase combination (1), which uses the L8 and C8 AltBOC(15,10) observables. These results are even more prominent in Figure 3c, which shows the 100 s TEC RMS for the studied combinations, which we selected as a noise proxy. The TEC noise for all studied combinations significantly increases at low elevation angles. At the same time, we can see that the TEC noise for the BeiDou AltBOC signals is one to two orders lower than for corresponding legacy signal combinations and is of the order 0.05 TECU at high elevations, which is very close to the results of the dual-frequency phase combination.

**Figure 4:**
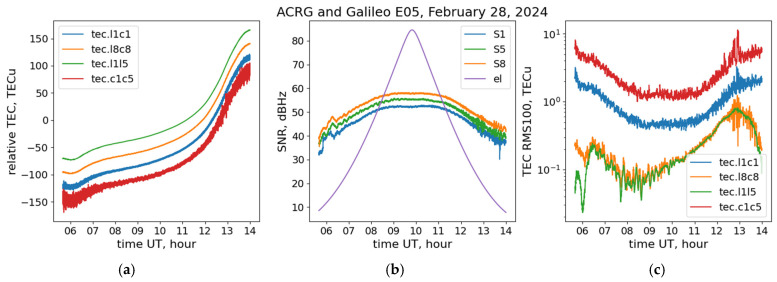
Slant TEC (**a**), SNR (**b**), and TEC RMS (**c**) for the ACRG-Galileo E05 pass on 28 February 2024. In panels (**a**,**c**), the green, orange, blue, and red lines correspond to L1L5, L8C8, L1C1, and C1C5 combinations, correspondingly. In panel (**b**), the orange, green, and blue solid lines correspond to S8, S5, and S1 observables, correspondingly. The purple line in panel (**b**) shows the satellite’s elevation angle.

We compared results obtained with the BeiDou AltBOC B2(a+b) signals with those obtained with Galileo AltBOC E5(a+b) at the same receiver. Figure 4 shows the results of such an analysis. The panels are arranged the same way as in Figure 3.

The results show the same features for the Galileo AltBOC signals that were observed for the BeiDou AltBOC signals. The SNR S8 for E5(a+b) almost reaches 60 dB-Hz for high elevations and similarly outperforms the SNR S1 for BPSK(1) and the SNR S5 for BPSK(10) by ~7.5 dB-Hz and ~2.5 dB-Hz, correspondingly. Note that the TEC noise figures for the single-frequency phase/code combinations of the BeiDou and Galileo AltBOC signals are also very close for the considered test passes. Note also that the presented results for the Galileo AltBOC correspond to those obtained in [31].

The presented case study gives only a hint of the overall AltBOC performance in TEC estimations. Thus, we statistically analyzed the whole considered dataset, involving all time intervals and all Galileo and BeiDou satellites. For Galileo, the results are shown in Table 3; and, for BeiDou, in Table 4, respectively. We considered three elevation intervals: low elevations (0–30°), medium elevations (30–60°), and high elevations (60–90°). The tables compare the averaged TEC RMS for two single-frequency (L8C8 and L1C1 for Galileo, and L2C2 for BeiDou) combinations and one dual-frequency (L1L5 for Galileo and L2L5 for BeiDou) combination for the three elevation intervals. Both the ionospheric irregularities and observables’ noises contribute to the 100 s *TEC RMS*. Thus, the results presented here are generally relevant to the undisturbed ionospheric conditions, when the influence of irregularities is relatively small.

The L8C8 single-frequency combination, associated with AltBOC modulation for both systems, consistently exhibits four-to-five-times-lower RMS values compared to the single-frequency combinations L1C1 and L2C2 built upon legacy signals. At the same time, it demonstrates only ~1.5-times-higher TEC RMS compared to the dual-frequency L1L5 and L2L5 phase combinations, generally at low elevation angles. At higher elevations, the single-frequency AltBOC combination, on average, provides the same level of TEC noise as the dual-frequency phase combinations for both systems. Note that the average AltBOC TEC noise is generally the same for both systems at all studied elevations ranges, with slightly higher values for BeiDou al lower elevations, which is likely to be due to the different orbits of the systems.

Overall, the AltBOC performance in the slant *TEC* makes this data type an excellent candidate to be adopted in algorithms of absolute vertical TEC estimations, like [12] and the one presented in the previous section. In fact, in vertical TEC estimation, data accumulation leads to noise reduction, so the effectiveness of such estimates does not depend crucially on quality (noise). However, it does depend crucially on the amount of input data, e.g., the number of satellites transmitting AltBOC signals. It is constantly increasing, currently numbering 26 for BeiDou and 25 for Galileo, which provides a sufficient amount of input data.

**Figure 5:**
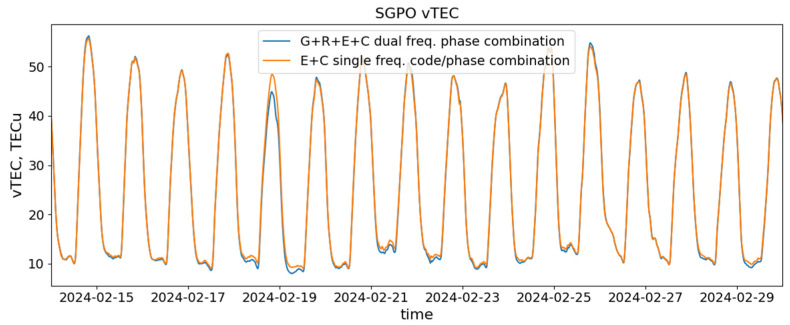
Absolute vertical TEC estimates at SGPO station with single-frequency combination of AltBOC signals from Galileo and BeiDou satellites (orange line) and from dual-frequency phase combination of non-AltBOC signals from GPS, GLONASS, Galileo, and BeiDou satellites (blue line).

We applied our absolute vertical *TEC* estimation algorithm to the whole considered period of observation at the SGPO station and compare the results obtained with the AltBOC and non-AltBOC signals. They are presented in Figure 5.

Two curves in this figure present the *vTEC* obtained with data only from the Galileo and BeiDou satellites transmitting AltBOC signals (using the single-frequency code/phase combination) and from the entire GNSS constellation, including GPS, GLONASS, Galileo, and BeiDou (using the dual-frequency phase combination of non-AltBOC signals). We can see that both estimates are in rather good agreement, typically within 1.5 TECU. Larger deviations, for example, on 18 February 2024, are mostly due to the geometry of the available IPPs and amount of data (satellite-to-receiver links) used in both cases, since the amount of AltBOC data is, on average, 2.5 times smaller.

If we would consider the dual-frequency *vTEC* estimates, limiting the constellation of satellites only to these, transmitting AltBOC, such deviations would almost completely disappear. This result, once again, demonstrates the high potential of AltBOC signals in ionospheric studies.

## 6. Performance of AltBOC Signals during Solar Radio Bursts

In this section, we present the results of the case study of the performance of the AltBOC signals under the conditions of natural RFI caused by SRBs on 28 August 2022 and 9 May 2024. The geomagnetic activity was quiet: during both events, the Kp index did not exceed 3.

SRBs are relatively short periods when solar radio emission is increased with respect to typical background levels. They commonly accompany solar flares and CME events. The SRB spectrum typically covers a wide band of frequencies but is not uniform. If an SRB occurs with significant emissions in the GNSS-band region and with a corresponding polarization (RHCP), it could result in RFI and affect the signals and the performance of the GNSS, which could, in turn, be detected in the SNR observables.

For the August 2022 event, Wright et al. [29] found a decrease in the SNR of legacy GNSS signals throughout the whole American sector. In our case study, we focused on AltBOC signals, and, thus, analyzed the high-rate data from the SGPO receiver, located in the mid-latitude American sector. SGPO records both E5(a+b) and B2(a+b) signals. Details about the station are given in Table 2.

In the case of the SRB event, the GNSS signal fading depends on the solar zenith angle at the station during observation and on the elevation of the satellite. The Galileo E34 and BeiDou C44 satellites were close to the zenith during the SRB event. We studied the SNR observables for AltBOC signals (S8) and its sidebands (S5 and S7), as well as for legacy signals S1 and S2, and compared them with the solar radio emissions observed at the Sagamore Hill observatory. Despite the strong effect of this SRB observed in the GNSS SNR, the Sagamore Hill radio observatory [38] recorded noise-like signals at 1415 MHz so we could not use these data, while it is the closest measured spectral line to the GNSS frequencies. It is the signature of the highly non-uniform spectra of radio emissions for this event. Thus, for the identification and timing of SRBs, we used the next closest 610 MHz line from this observatory.

**Figure 6:**
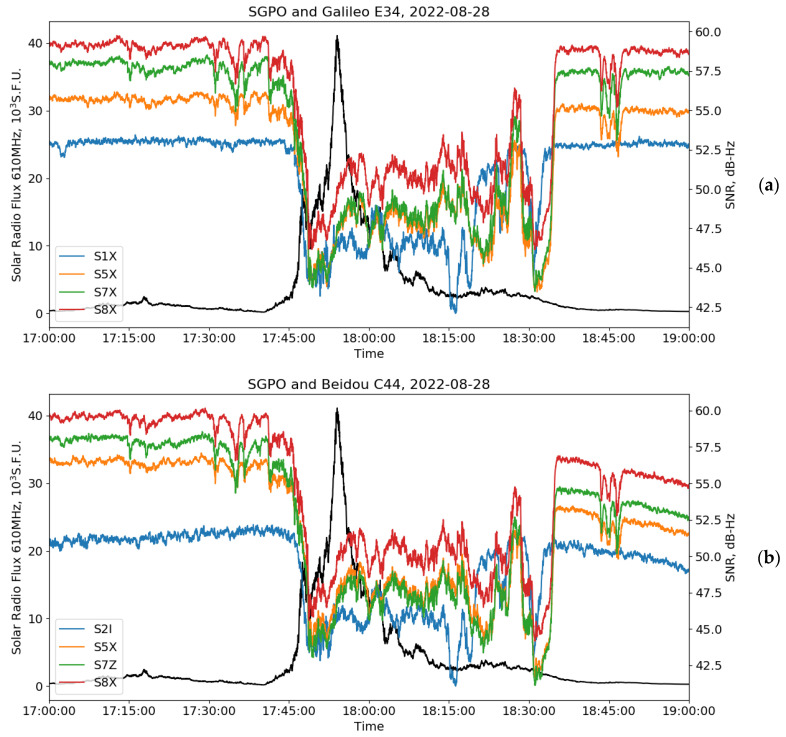
Signal-to-noise ratio (SNR) from the Galileo E34 (**a**) and BeiDou C44 (**b**) satellites observed at the SGPO station on 28 August 2022, alongside the corresponding solar radio flux at 610 MHz (black curves). In panel (**a**), the red line shows the SNR S8X of E5(a+b) signal, the green line represents the SNR S7X of E5b sideband, the orange line—SNR S5X of E5a sideband, and the blue line—the SNR S1X of E1 signal; and, in panel (**b**), the red line shows the SNR S8X of B2(a+b) signal, the green line—the SNR S7Z of B2b sideband, the orange line—SNR S5X of B2a sideband, and the blue line—the SNR S2I of B1I signal.

The obtained results are shown in Figure 6 for the E34 (a) and C44 (b) satellites. We observe a rapid increase in the solar radio emission starting at ~17:45 UT and exceeding 40,000 s.f.u. (indicating an SRB event). The fading of the Galileo and BeiDou signals covers the period of ~1 h—which is twice as long as the period when we observed increased solar radio emission at 610 MHz. It supports the conclusion on the highly non-uniform spectrum of the studied SRB. Before the disturbance, we observe significantly lower (up to 10 dB-Hz) SNRs for the non-AltBOC signals E1 and B1I compared to the AltBOC ones. As the SRB evolves, we can observe fading (SNR decrease) for all considered GNSS signals, both for BeiDou and Galileo.

We can also notice that this fading is not equal for all studied signals. For the E1 and B1I signals, this fading is smaller (5 dB-Hz and even more) than for the AltBOC E5 and B2 signals and their sidebands. At the same time, the SNRs of all signals do not test down the typical ~20–25 dB-Hz threshold established for loss-of-lock events. The reason for such a different fading of AltBOC and non-AltBOC signals could be in the weaker RFI mitigation for AltBOC signals during this peculiar event, which is not a property that could be expected from AltBOC signals.

Another possible explanation is, once again, the non-uniform spectrum of this SRB. We should take into account that E1 and B1I from one side and E5a, E5b, E5(a+b), B2a, B2b, and B2(a+b) from another occupy rather different parts of the spectrum, near 1570 MHz for the first ones and near 1190 MHz for the last ones. We can observe a high coherence of SNR variations between the E1 and B1I signals and their rather significant difference with the E5 and B2 SNR variations, which, in turn, look highly coherent with each other. These peculiarities are most prominent around 18:00 UT, 18:15 UT, and 18:30 UT, as well as before and after the main phase of SRB. This could suggest the conclusion that RFI (SRB emission) was larger around 1190 MHz than around 1570 MHz, but we cannot support it since no independent measurements of the solar radiation spectrum covering those bands were available. Another possible explanation is the processing of different signals in the receiver. To test it, one should conduct further investigations with different models of receiving equipment, which realize different signal processing schemes for AltBOC, which is beyond the scope of current paper.

**Figure 7:**
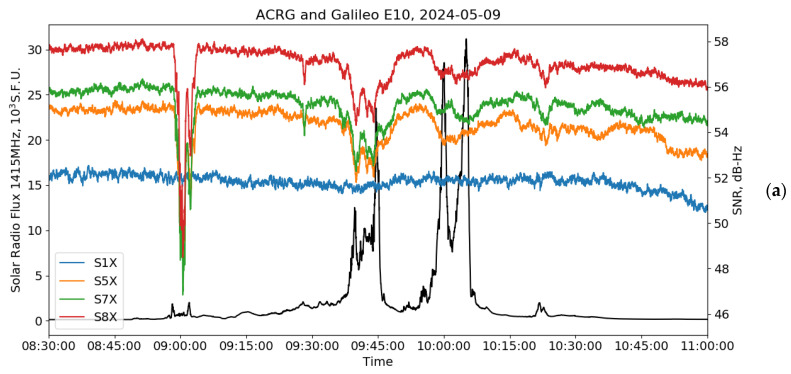

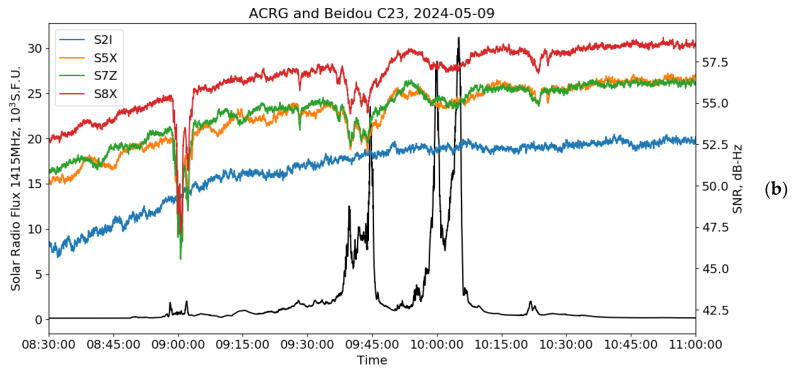
Signal-to-noise ratio (SNR) from the Galileo E10 (**a**) and BeiDou C23 (**b**) satellites observed at the ACRG station on 9 May 2024, alongside the corresponding solar radio flux at 1415 MHz (black curves). In panel (**a**), the red line shows the SNR S8X of E5(a+b) signal, the green line—the SNR S7X of E5b sideband, the orange line—SNR S5X of E5a sideband, and the blue line—the SNR S1X of E1 signal; and, in panel (**b**), the red line shows the SNR S8X of B2(a+b) signal, the green line—the SNR S7Z of B2b sideband, the orange line—SNR S5X of B2a sideband, and the blue line—the SNR S2I of B1I signal.

Figure 7 supports the previous results on signal fading under solar radio bursts. The recent 9 May 2024 SRB was recorded using the data of the 1415 MHz channel of the San Vito observatory [38]. During this event, we used data from the ACRG receiver, which was on the sunlit side of the Earth. Again, we observe that AltBOC signals are vulnerable to SRBs. It is surprising that weaker SRB fluxes resulted in a stronger SNR drop: 10 dB-Hz when the flux was <5000 s.f.u. vs. 1–2 dB-Hz when the flux exceeded 30,000 s.f.u. We suppose that it is an effect of the peculiarities in the solar radio emission spectrum. Most probably, the weaker SRB at 1415 MHz was stronger in the Galileo/BeiDou band (~1200 MHz). In any case, both systems show the same pattern and both show a significant difference with legacy Galileo E1 and BeiDou B1I. This supports a hypothesis that a wider AltBOC signal frequency bandwidth increases the integral noises and deteriorates the tracking phase-locked loops.

## 7. Discussion and Conclusions

Our research shows the advancement that AltBOC signals provide for the TEC estimation. There are many factors that deteriorate the TEC quality [39], while only few factors increase it: an increase in the satellite transmitter power [28], an enhanced antennae, or advanced signal coding. The current study focuses on the latest point and provides a comprehensive evaluation of the performance of BeiDou B2 and Galileo E5 signals, which implement advanced AltBOC coding, particularly focusing on their SNR and applicability for the TEC estimation under various ionospheric conditions.

Our results indicate that the TEC RMS for the single-frequency combination of the BeiDou B2 signal is comparable to that of the Galileo E5 signal. The results reveal that the noise level of the single-frequency AltBOC TEC is very close to that of the dual-frequency phase combination, especially at higher elevation angles (60–90°), where the noise is as low as 0.09 TECU. This finding is fully consistent with results obtained previously for Galileo [31] and significantly broadens the scope of the single-frequency TEC combination, making it a reliable alternative to the dual-frequency phase combination for high-precision ionospheric monitoring.

Our analysis also shows that, under undisturbed conditions, the SNR for E5 and B2 signals is generally higher by approximately 7.5 dB-Hz compared to E1 and B1 signals. This higher SNR translates into a better signal quality and robustness, which is essential for high-precision ionospheric monitoring. However, during the studied SRB event, we observed a significant fading for AltBOC-modulated E5 and B2 signals, exceeding those for E1 and B1I BPSK-coded signals. This highlights the possible susceptibility of even advanced modulation schemes like AltBOC to intense space weather events and raises questions for further studies. Nevertheless, an initially higher SNR in undisturbed conditions could prevent AltBOC signals from breaking down the typical 25–30 dB-Hz loss-of-lock (LoL) threshold. This suggests that AltBOC signals could maintain a high reliability under extreme space weather conditions. That can be used for suggesting new techniques for precise navigation [40].

A promising trend observed in this study is the increasing number of satellites transmitting AltBOC signals, currently numbering 26 for BeiDou and 25 for Galileo. This expanding constellation enhances the reliability of the single-frequency *vTEC* estimation and supports the continued development and improvement of GNSS-based ionospheric studies.

In conclusion, our findings not only highlight the robustness and effectiveness of the AltBOC modulation in GNSS signals but also suggest that, as more satellites equipped with AltBOC signals are deployed, the accuracy and reliability of single-frequency GNSS applications will continue to improve. This makes AltBOC modulation a promising choice for future ionospheric research and navigation technologies, providing a solid foundation for addressing the challenges posed by complex space environments.

## Figures and Tables

**Table 1:** Main characteristics of the Galileo E5 and BeiDou B2 signal.

System	Signal	Notation in RINEX 3.05	Central Frequency, MHz	Minimum Bandwidth, MHz	Modulation Type	Chip Rate, MHz
Galileo	E5a	C5X, L5X, D5X, S5X	1176.45	20.46	BPSK(10)	10.23
	E5b	C7X, L7X, D7X, S7X	1207.14	20.46	BPSK(10)	10.23
	E5 (E5a + E5b)	C8X, L8X, D8X, S8X	1191.795	51.15	AltBOC(15,10)	10.23
BeiDou	B2a	C5X, L5X, D5X, S5X	1176.45	20.46	QPSK(10)	10.23
	B2b	C7D, L7D, D7D, S7D	1207.14	20.46	BPSK(10)	10.23
	B2 (B2a + B2b)	C8X, L8X, D8X, S8X	1191.795	51.15	AltBOC(15,10)	10.23

**Table 2:** Receiving sites.

Parameter	Value	Value
Station Name	SGPO	ACRG
Coordinates	36.604° N, 97.485° W	5.603° N, 0.187° W
Location Name	Billings, OK, USA	Accra, Ghana
Receiver Type	Javad TRE_3S	Javad TRE_3S
Signals Tracked by Receiver	GPS, GLO, GAL, BDS, SBAS	GPS, GLO, GAL, BDS, IRNSS, SBAS
Antenna Type	JAVAD RingAnt-G5T	SEPTENTRIO POLANT CHOKE RING B3/E6
Data Rate	1 Hz	1 Hz

**Table 3:** Galileo average *TEC RMS* under quiet conditions.

Elevation, °	L8C8 RMS, TECU	L1C1 RMS, TECU	L1L5 RMS, TECU
0–30	0.25	0.88	0.19
30–60	0.14	0.63	0.13
60–90	0.09	0.47	0.08

**Table 4:** BeiDou average TEC RMS under quiet conditions.

Elevation, °	L8C8 RMS, TECU	L2C2 RMS, TECU	L2L5 RMS, TECU
0–30	0.26	0.87	0.22
30–60	0.15	0.60	0.14
60–90	0.09	0.45	0.09

## Data Availability

The RINEX data used in the study are available at https://cddis.nasa.gov/archive/gnss/data/ (accessed on 1 June 2024).
